# Risk and protective factors for psychotherapists in regions of crisis: what do psychotherapists need to face the danger of secondary traumatization in Kurdish Iraq?

**DOI:** 10.3389/fpubh.2026.1959666

**Published:** 2026-10-13

**Authors:** Gabriel Kornwachs, Martin Hautzinger, Jan Kizilhan

**Affiliations:** 1 Department of Clinical Psychology and Psychotherapy, University of Tübingen, Tübingen, Germany; 2 Institute for Transcultural Health Research, Duale Hochschule Baden-Württemberg, Stuttgart, Germany

**Keywords:** consequences of war, indirect trauma, mental hygiene, middle east mental health, psychological wellbeing of helpers, PTSD, secondary trauma, survivors of trauma

## Abstract

Transgenerational and secondary traumatization are key aspects to be addressed when aiming for sustainable psychosocial support in post-war regions. Within this context, the primary goal of the Institute for Psychotherapy and Psychotraumatology (IPP) is to establish sustainable structures for psychotherapy, acknowledging that psychological distress does not simply vanish with the end of a war. Rather, it only becomes fully visible when considering not only the consequences for beneficiaries but also for helpers and subsequent generations. Because mental health workers themselves face a high risk of suffering from secondary traumatization, deliberate measures must be implemented to ensure that helpers do not become secondarily traumatized during and/or after their service. Using the IPP as a community case study, this article analyzes both general and context-specific risk factors for its students and local staff. Furthermore, it identifies universal protective factors that mitigate these risks and outlines culturally specific protective factors against secondary trauma. It also presents critical reflections on how a far-sighted and realistic understanding of cultural dynamics can contribute to the systemic protection of aid workers. Ultimately, the article suggests that providing helpers with comprehensive psychosocial support, physical and operational safety, structured peer support, transparent financial frameworks, clear future career prospects, and institutional recognition of their work are of paramount importance in sustaining long-term psychological wellbeing for psychotherapists.

## 1 Introduction

Traumatization is the process through which exposure to an overwhelmingly distressing event outstrips an individual's internal and external coping mechanisms, resulting in lasting psychological, emotional, or physiological harm. While “trauma” refers to the event or the wound itself, “traumatization” is the dynamic process of becoming traumatized and how that wound integrates into a person's life (1).

In the field of psychotraumatology, transgenerational trauma refers to the transmission of psychological, behavioral, and physiological consequences of severe trauma to subsequent generations who did not directly experience the original event. This complex process involves interacting psychosocial and biological pathways. Behavioral transmission is strongly influenced by family environment, attachment patterns, and parenting practices. Parents with unresolved post traumatic stress disorder (PTSD) or complex trauma may develop altered parenting styles characterized by hypervigilance, emotional unavailability, overprotection, or controlling behaviors (2). Trauma-related communication patterns also contribute to transmission (such as family silence, or age-inappropriate over-disclosure) which may expose children to secondary traumatization (3). Research has increasingly focused on biological pathways, including epigenetic modifications such as DNA methylation, which influence gene expression without altering the DNA sequence (4). Epigenetic changes affecting stress-regulation systems, including the HPA axis, may contribute to increased stress vulnerability and susceptibility to psychiatric disorders in subsequent generations (2, 4).

Secondary traumatization represents the indirect psychological distress experienced by professionals through their exposure to the traumatic experiences of others (5). It can occur when a helper absorbs the trauma narratives, imagery, and pain of survivors, leading to ongoing symptoms. The term often describes the broader process or end-result of actually developing trauma symptoms due to indirect exposure, whereas secondary traumatic stress (STS) is defined as the stress response, such as intrusive thoughts, or fatigue, that happens after a person hears about or witnesses another person's trauma. STS mimics Post-Traumatic Stress Disorder (PTSD) and often appears quickly after a specific event. Known as a stress response triggered by supporting or being close to individuals dealing with trauma, STS regularly affects frontline healthcare professionals such as doctors, nurses, psychologists, social workers, and humanitarian aid workers.

Thus, transgenerational and secondary trauma are both forms of indirect trauma in which an individual exhibits symptoms of trauma, such as hypervigilance, anxiety, or mood dysregulation, without having directly experienced the original traumatic event itself. Both conditions stem from exposure to another person's severe suffering, meaning that the affected individual is responding to an external event mediated through another person (6, 7). Despite these psychological overlaps, the two phenomena differ fundamentally in their transmission mechanisms, relationships, and temporal contexts. Secondary trauma occurs in the present day, primarily through direct empathic engagement with trauma survivors, and commonly affects healthcare workers who actively listen to or witness the graphic details of a victim's firsthand trauma (5). Conversely, transgenerational trauma is transferred across ancestral lines, typically moving from a trauma-surviving parent or grandparent to their descendants (3). Furthermore, individuals experiencing secondary trauma are generally aware of the specific stories or clients causing their distress, whereas those affected by transgenerational trauma often experience diffuse, unexplained feelings of shame, grief, or vulnerability without connecting them to a specific event in their personal biography [e.g., (8)]. Key differences between transgenerational and secondary trauma are described in Table 1.

**Table 1:** Differences between secondary and transgenerational trauma and examples within the IPP context.

Dimension	Secondary trauma	Transgenerational trauma
Relationship Context	Often professional or therapeutic (e.g., therapist-client, first responder-victim).	Familial and ancestral (e.g., parent-child, grandparent-grandchild)
Transmission mechanism	Present-day empathetic exposure to graphic details of another person's trauma.	Combined psychosocial dynamics (family silence, parenting styles) and epigenetic inheritance.
Temporal dynamic	Occurs in the current lifespan through immediate interaction.	Accumulates and unfolds across multiple generations over decades
Awareness of cause	Affected individuals usually know which specific case or story triggered their distress.	Descendants often experience diffuse, unexplained distress without a clear biographical cause
Example within IPP context	IPP student hear stories of atrocity within their everyday profession. Survivors are sharing their story with IPP students	IPP students are themselves sometimes children of victims of violence. Some IPP students are not only facing family silence toward their history of the experienced trauma, but even more they are now facing a rather unaccepting or even hostile attitude of society toward psychotherapy as a profession, as it treats “the weak.”

## 2 Background and purpose of the IPP

The “Institute for Psychotherapy and Psychotraumatology” (IPP) was established in 2016 at the University of Duhok. It addresses the severe lack of psychotherapeutic services in the Kurdistan Region of Iraq (KRI), which hosts approximately one million refugees and internally displaced persons (IDPs), many of whom were traumatized by the so-called Islamic State [ISIS; (9)]. Although the KRI is not currently the main theater of active warfare, its society, economy, and institutions remain profoundly shaped by regional instability and the legacy of war [e.g., (10)]. Consequently, research from (post)-war regions is highly applicable to the KRI. While a “region of conflict” is defined as the actual territory of active combat, a “region of war” marks the broader perimeter where military actions are possible. This article expands its scope to include areas suffering from the long-term consequences of war, such as neighboring regions facing a surge in internally displaced persons (IDPs).

The IPP offers a long-term, 3-year master's program to train professional local psychotherapists, which consists of a comprehensive theoretical component, as well as practical clinical training with professional and individualized support for the therapist via supervision. The program's content and teaching methods were primarily developed to meet the urgent needs of regions of crisis and its specific location. Consequently, Kurdish culture heavily influences and shapes both the academic material and the instructional approach. To ensure the program constantly adapts and improves, the founders and local staff hold regular reflection meetings. Today, these updates are managed almost entirely by the local staff to best serve the evolving needs of the students.

To date, the IPP has graduated 69 students (31 female, 38 male) between the ages of 29 and 55 (average age: 36). Currently, an additional 40 students are approaching graduation or have completed a substantial portion of the master's program. To ensure the project's autonomy and sustainability, international experts are gradually handing over teaching and organizational duties to a core group of local graduates. It is believed that local lecturers are best suited to adapt evidence-based Western methods to the local language, culture, and religious norms. Thus, a step-by-step transition process is currently underway, ensuring that responsibilities are transferred in phases: moving from supporting international experts to taking full responsibility for lectures, supervision, and curriculum organization. Within this process, the local staff/psychotherapists face many challenges, which may undermine their psychological wellbeing. This article examines these specific challenges, relates them to established risk factors for psychotherapists, and discusses potential strategies to mitigate these risks while emphasizing the importance of protecting the psychological wellbeing of helpers in the region.

Furthermore, the IPP aims to establish long-lasting structures, recognizing that psychological distress also affects subsequent generations. Without a permanent and locally implemented and operated psychotherapeutic infrastructure, the cycle of traumatization within Kurdish and Yazidi society cannot be broken (11). Transgenerational and secondary traumatization need to be addressed, when striving to improve a society's mental health over the long term. Consequently, helpers need to find acceptable working conditions under which they provide psychosocial care (including psychological support and social/environmental support). Using the IPP as a practical example, general and culturally specific risk and protective factors are discussed in the next section. Since secondary and transgenerational trauma in the Middle East is frequently processed within familial and collective contexts rather than solely on an individual level, a profound understanding of local norms and identities and the cultural adaptation of Western therapeutic models is essential both for the establishment of a functioning program and for the successful treatment of (intergenerational) trauma. Respecting cultural and social norms while also challenging them when necessary, rather than naively ignoring cultural challenges, helps when finding practical solutions (12–14). Despite the importance of both transgenerational and secondary traumatization, this article focuses primarily on the latter, mainly because of its high everyday relevance to the psychotherapists working for the IPP.

Since mental health workers face a high risk of secondary trauma, it is essential to ensure that those providing help do not also suffer secondary traumatization. Situational and culturally specific risk factors can vary and therefore need to be addressed within an individual, context-specific approach. As a consequence, this article will discuss the general but also culturally specific risk factors for secondary traumatization of helpers within the context of the practical example of the implementation of the IPP in the Kurdish Region of Iraq. It discusses specific risk factors affecting the project's staff. Within the context of Kurdish culture, it also provides examples of cultural-specific protective factors, considering how a far-sighted and realistic understanding of cultural elements can contribute to the protection of helpers. Psychosocial support, safety, peer support, clear financial structures, future prospects, and appreciation for their work appear to be of particular importance in this context. Ultimately, the article proposes practical strategies to reduce risk factors for secondary traumatization among the IPP's local staff, including students, therapists, and lecturers.

## 3 Risk factors for secondary traumatization

### 3.1 General risk factors

A high volume of traumatized clients and extensive hours spent conducting deep trauma inquiries or listening to graphic details are associated with secondary traumatic symptoms (15, 16). Besides the high workload and intensity of exposure, weak institutional structures, deficient peer support networks, unsupportive supervision, and an absence of formal outlets to debrief or process raw emotional encounters act as massive organizational catalysts for stress (15, 17).

Furthermore, individual psychological vulnerabilities contribute to secondary trauma. Psychotherapists with a personal or prior history of direct trauma are statistically much more vulnerable to secondary traumatization when managing client files (18). Low self-differentiation (the inability to separate one's own emotional life from a client's) also increases the risk (16). Maladaptive coping strategies, such as relying heavily on cognitive avoidance, emotional suppression, or self-isolation maintain and worsen symptoms over time compared to active or compassion-focused coping (19).

Lastly, certain demographic factors can increase the risk of secondary trauma. Meta-analyses show that younger age, less professional experience, and female sex are frequently associated with elevated baseline risks for post-traumatic and secondary traumatization (17, 18). It is worth mentioning in this context, that nearly half of the IPP students are female, and most are under 30 years of age. These general risk factors for secondary traumatization are summarized in Table 2.

**Table 2:** General risk factors for secondary trauma.

Risk factor category	Description/Impact	References
High workload and exposure intensity	High volumes of traumatized clients and long hours listening to graphic details or conducting deep trauma inquiries drive secondary traumatic symptoms.	(15, 16)
Lack of organizational support	Weak institutional structures, unsupportive supervision, inadequate peer networks, and a lack of formal debriefing outlets catalyze stress.	(15, 17)
Individual psychological vulnerabilities	Personal history of prior trauma significantly increases vulnerability during file management. Low self-differentiation further heightens risk.	(16, 18)
Maladaptive coping strategies	Dependence on cognitive avoidance, emotional suppression, and self-isolation worsens long-term symptoms relative to active coping.	(19)
Demographic factors	Younger age, less professional experience, and female sex correlate with elevated baseline risks.	(17, 18)

### 3.2 Specific risk factors for psychotherapists in war regions

When psychotherapists operate within active war zones or acute humanitarian crises, unique systemic, environmental, and physical stressors substantially increase the risk (20). Working amidst ongoing gunfire, mortar shelling, landmines, or threats of abduction introduces an active layer of primary existential dread that deeply erodes psychological resilience against secondary trauma (17, 21). Logistical deprivation and field hardships, e.g., the extreme physical demands of a combat zone, the severe lack of sleep, poor nutrition, physical pain from overexertion, and complete breakdown of reliable infrastructure or communication - severely compromise the physical body's capacity to process cognitive and emotional trauma (16, 17). As resources are often severely limited in war zones, helpers must routinely navigate complex, highly agonizing moral dilemmas (e.g., strict triage rules, refusing care due to supply depletion, or working with highly inadequate equipment), which can trigger profound feelings of helplessness and ethical failure (17, 22).

These extreme conditions often have to be faced by personnel, that face inadequate role training. Due to sudden surges of mass violence, local individuals are frequently recruited rapidly into humanitarian frontline roles (such as distributing food, translation, or basic case management) with little to no formal psychological training, leaving them completely unprotected against heavy traumatic disclosure (23). Furthermore, local or national humanitarian helpers often belong to the exact same population they are serving (23). They do not just listen to trauma; they are simultaneously surviving the same war, shelling, displacement, and loss of loved ones alongside their clients, creating a dangerous layering of primary and secondary trauma (21, 23).

#### 3.2.1 Recent security developments related to the Iran–US conflict in 2026

Between February and May 2026, escalating regional tensions and attacks by Iranian militias affected the Duhok Governorate, including threats against Western-linked institutions in the Kurdistan Region. Although the University of Duhok (UoD) did not suspend operations, students experienced significant indirect consequences. Drone attacks near Duhok, ongoing security alerts (24), and the proximity of military bases contributed to an atmosphere of persistent insecurity, causing anxiety, distraction, and uncertainty among students. Attendance fluctuated during late March and April 2026, as some students chose to remain at home or return to safer rural areas. Increased checkpoint controls, heightened security measures, and commuting difficulties further disrupted daily routines. As a precautionary measure, the IPP's outpatient psychotherapy clinic reduced its working hours. In addition, the curriculum had to be adapted temporarily: lectures were moved online, and regular online supervision was severely affected by unstable internet connections (which were based on observations by the authors and complemented by direct reports of students via supervision and staff meetings). Overall, these developments seem to have contributed to a profound sense of insecurity among many IPP students and staff. Kizilhan et al. (11) describe how disrupted infrastructure (e.g., electricity, internet, and restricted transport) compounds psychological distress and limits clinical continuity in the Duhok Governorate. Furthermore, the authors describe how active regional conflicts and security instability create chronic secondary traumatic stress for local communities and clinical/academic personnel. Specific risk factors for secondary trauma for psychotherapists in war regions are summarized in Table 3.

**Table 3:** Specific risk factors for secondary trauma for psychotherapists in war regions.

Risk factor category	Description/Impact	Key citations
Direct threat to personal safety	Operating under active gunfire, shelling, landmines, or kidnapping threats introduces constant primary existential dread that wears down psychological resilience.	(17, 21)
Logistical deprivation and field hardships	Extreme physical demands, sleep deprivation, poor nutrition, physical strain, and infrastructure breakdown impair the body's ability to process trauma.	(16, 17)
Moral injury and ethical dilemmas	Severe resource constraints force agonizing choices (e.g., triage decisions, care refusal, sub-standard equipment), driving deep feelings of helplessness and ethical failure.	(17, 22)
Inadequate role training	Rapid recruitment of local individuals into frontline roles during mass violence surges without formal psychological training leaves them unprotected against trauma.	(23)
Shared traumatic reality	Helpers undergo the same active threats, displacement, and loss as their clients, creating stacked primary and secondary trauma	(21, 23)

## 4 Culturally specific risk factors (example of Kurdish culture)

Because local helpers often share the same historical and cultural background as their clients, it is assumed that their vulnerability to absorbing secondary trauma might be compounded by distinct cultural effects. The following aspects are primarily drawn from the authors' experiences and discussions with (former) students at the IPP. Thus, the authors' supervision and teaching experience at the IPP are the main base for the following points. The insights were informally gathered through meetings, supervision sessions, lectures, and discussions over the years. The observations stem from informal impressions of the authors. As for these informal impressions no additional consent was obtained, all potentially identifying details were carefully removed to preserve anonymity. To maintain a clear focus on the clinical and systemic impacts of secondary traumatization, this section avoids a deeper linguistic or ethnographic analysis of local terminology.

### 4.1 Over-identification & shared cultural trauma (*Hevpişkinî*)

The transcultural concept of Hevpişkinî, representing communal solidarity and shared destiny within Kurdish communities, is defined and analyzed through shared empathy and cultural frameworks (25). Thus, this concept might be both a risk and a protective factor. Unlike international aid workers, local Kurdish helpers frequently share a trauma history themselves (e.g., surviving the Anfal campaign, chemical attacks, or displacement by ISIS) (11, 26). This may lead to blurred boundaries: When a client narrates experiences of war or loss, it serves as a powerful psychological trigger for the helper's own potentially unhealed or transgenerational wounds, dismantling the cognitive distance required to prevent secondary traumatization (11). Whereas, the sense of shared experience might be a protective factor against traumatization (13), a strong sense of community might also foster the risk of secondary traumatization.

### 4.2 Collective burden and the downside of family collectivism (*Xêzan*)

While the extended family is a protective shield, it can also become a vector for secondary traumatization. In a collectivistic society, a helper does not just bear the weight of their formal clients; they are culturally obligated to absorb and solve the psychological and economic distress of their own relatives (28). This leads to indirect stress via proximity. The constant, dense socialization within the extended family deprives the helper of quiet, individualized spaces to process and discharge emotional residue (12). The much-needed privacy and safe space for helpers for their own decompression often do not exist.

### 4.3 The cultural duty of infinite sacrifice (*Fidakarî*)

Cultural expectations place immense moral pressure on helpers to act with absolute self-sacrifice (*Fidakarî*) for the survival of the community. Establishing necessary professional boundaries - such as refusing to take calls after hours or limiting caseloads - is often viewed culturally as a betrayal of solidarity or a lack of compassion. This may lead to internalized guilt for helpers and cause helpers to override their own psychological limits until complete emotional collapse occurs.

### 4.4 Severe societal stigma and spiritual shame (*Serm*)

Deep-seated societal stigma surrounding mental health issues means that local helpers who show signs of secondary trauma risk facing condemnation or being labeled as “weak” by family and peers (27). This projection of weakness may mislead helpers into suppressing their symptoms. If a helper fears losing social standing or being perceived as lacking faith, they may suppress their symptoms. By suppressing intrusive thoughts, anxiety, or depressive symptoms, helpers may hide their struggles to protect their social standing. This suppression may accelerate the transition from mild distress to severe secondary PTSD.

### 4.5 Inter-tribal or inter-religious countertransference

The KRI hosts complex sociopolitical and religious realities involving different tribal lineages, political factions, and minorities (e.g., Yazidi survivors, Syrian Kurdish refugees, Arab IDPs) (11, 28). Helpers may experience profound internal conflict or moral injury when caring for survivors of specific subgroups if their own family historically clashed with that group, or conversely, if they feel paralyzed by the overwhelming historical guilt of what their community went through (11).

### 4.6 Facing moral dilemmas

Case supervisions and student reports reveal that students sometimes confront a difficult moral dilemma when assigned to treat former ISIS terrorists. Substantial emotional and psychological stability is required to treat these patients, especially when relatives or friends have been victims of the atrocities of ISIS members. On the one hand, the own emotional rejection is a natural human reaction in such a case. On the other hand, therapists learn that everyone deserves psychological treatment. Of course, taking over such a case is always voluntary and should be guided by close supervision that allows the therapist to share their own emotional experience. The latter illustrates the vital importance of quality supervision, peer support, and a healthy working environment for support staff. As described above, some of the aforementioned risk factors strongly apply to our (former) students. The project-specific additional risk factors will be described in section 6.2.

## 5 Risk-alleviating factors

General protective factors against secondary traumatization include organizational support, cognitive coping strategies, effective supervision, and physical self-care. These factors have consistently been associated with lower levels of secondary traumatic stress [e.g., (29)]. A longitudinal study of 400 UK healthcare professionals demonstrated that perceived workplace support - including collegial support, visible leadership, and managerial concern significantly reduced depression, insomnia, emotional exhaustion, and poor wellbeing (30). These findings underline the importance of supportive working environments for psychotherapists.

### 5.1 General protective factors for psychotherapists

On an individual level, practitioners should be encouraged to utilize cognitive reframing, as deploying adaptive coping mechanisms and cognitively reappraising traumatic events - rather than relying on avoidance strategies - serves as a powerful cognitive shield to protect long-term mental health (31). Additionally, establishing absolute role clarity by ensuring clearly defined and contained job responsibilities substantially reduces operational ambiguity, which directly aids professionals in maintaining strict, healthy boundaries between their demanding professional duties and private life (32, 33). This structural foundation must be reinforced by proactive managerial support, as visible and accessible leadership that provides active recognition for helpers has been shown to impact job satisfaction and reduce burnout among frontline helpers (34, 35).

Furthermore, structured peer exchange mechanisms play a vital role in psychological stabilization, which is achieved through regular intervision sessions that allow equals to discuss complex cases without hierarchical pressure, as well as formal and informal debriefings immediately following critical incidents to help process traumatic material (5). This professional validation provides indispensable psychological stability that informal private circles typically cannot offer. To alleviate the heavy individual guilt often associated with trauma work, institutions must cultivate an explicit learning culture over a punitive blame culture by establishing safe, sanction-free reporting systems for critical incidents that validate human fallibility and treat procedural errors as collective opportunities for systemic improvement (36).

Developing a strong community network can be used as a primary protective barrier against professional isolation, allowing aid workers to experience a profound sense of belonging within a larger organizational structure (37). Within these networks, a shared emotional burden normalizes distressing psychological reactions, while comprehensive resource pooling ensures immediate access to peer assistance, shared expertise, and logistical support during acute crises (36).

Finally, shifting the institutional focus toward realistic professional competence rather than establishing an unrealistic picture of being a healer or savior protects helpers from chronic exhaustion. This paradigm shift requires discarding the myth of the flawless hero, embracing continuous skill improvement through specialized training, and actively accepting personal boundaries of capacity and expertise (38). Psychotherapists must acknowledge the limits of treatment. Especially for young therapists, it is crucial to accept that every patient has the right to make their own decisions regarding treatment (as long as there is no immediate risk to themselves or others), including the decision not to change. Therapists may offer support and guidance, but they must also accept that patients are not always ready or willing to accept the help offered.

Combined with classic mental health self-care strategies, such as strict boundaries between work and private life, proactive physical self-care, and regular external clinical supervision, these comprehensive measures effectively foster long-term psychological resilience in high-stress environments (5). To effectively mitigate the compounding effects of secondary trauma among local practitioners in the Kurdistan Region of Iraq (KRI), organizations must establish robust institutional frameworks centered on community support, peer exchange, constructive error tolerance, and specialized competence.

### 5.2 Culturally specific protective factors (example of Kurdish culture)

In the Kurdistan Region of Iraq, psychosocial work (providing psychological and social support to help individuals cope with mental, emotional, and environmental challenges) and mental hygiene are profoundly shaped by distinct sociocultural, historical, and religious dimensions. Local helpers, who are frequently members of the very communities affected by decades of conflict, can draw upon traditional cultural mechanisms as important resilience factors. The mentioned Islamic-Arab shaped concepts and their cultural narratives and patterns of interpretation (in particular) are partly also adopted by members of religious minorities, including Christians and Yazidis, or used in a functionally similar way.

#### 5.2.1 Collective resistance (Berxwedan)

A foundational element of this cultural coping framework is collective resilience and a shared historical survival consciousness, often encapsulated by the Kurdish concept of *Berxwedan* (resistance). This mindset transforms passive suffering into an active mechanism of survival, allowing psychotherapists to frame their demanding labor not merely as a job, but as a meaningful contribution to the historical preservation and endurance of their people. Secondary traumatization often shatters a helper's worldview, inducing feelings of helplessness. The cultural framework of *Berxwedan* might help reframe the survivors' suffering - and the helper's exposure to it - as an active, honorable act of defiance and community preservation. This high level of shared group identification may help the helper find immediate cognitive meaning, which might shield them from absorbing the trauma as a purely destructive force. This transgenerational strength actively reframes historical atrocities, such as the Anfal campaign or the attack of ISIS, passing stories through generations as structural evidence of the community's innate psychological durability rather than viewing them strictly as a source of ongoing trauma.

#### 5.2.2 The unity of family (Xêzan)

STS thrives on social isolation (39). Helpers often feel their private networks cannot comprehend the horrors they witness. In Kurdish culture, the unconditional, non-clinical backing of the extended family serves as a natural emotional filter (12). The extended family unit (*Xêzan*) can operate as a primary and indispensable source of psychosocial safety. By immediately reintegrating the helper into a secure, collective family dynamic upon returning home, the family structure acts as an active counter-measure to the alienation and emotional numbing characteristic of secondary trauma (12). Therefore, in the Kurdish context, mental hygiene does not manifest solely through formal, Westernized therapeutic structures or professional supervision. Instead, the expression and sharing of professional or personal distress occur organically within extended family systems that offer unconditional structural backing. This collective distribution of emotional burdens can significantly diminish the threat of individual burnout and psychological isolation among local helpers. Therapists may share stories anonymously, provided that strict confidentiality is maintained. It is crucial, however, that the information shared contains no identifying details that would allow the recipient to discover the patient's identity.

#### 5.2.3 The belief in destiny (Qadar)

In Islam, the concept of *Qadar* (often translated as Divine Decree or Predestination) represents God's absolute sovereignty, omnipotence, and His infallible foreknowledge of all events [e.g. (40)]. Interestingly, these narratives and interpretive frameworks are sometimes adopted or used in functionally similar ways by members of religious minorities in the region, including Christians and Yazidis. However, this can lead to misconceptions. The Yazidi cosmology- which centers on *Xwedê* (God), *Tawûsî Melek* (the Peacock Angel), and its own distinct understanding of fate and providence - should not be easily or superficially subsumed under Islamic concepts.

A core symptom of secondary traumatization is existential dread and hyperarousal caused by an overwhelming sense of responsibility for the suffering of others. Spiritual alignment through *Qadar* (destiny) might allow Kurdish helpers to psychologically externalize a portion of this existential burden. Believing that ultimate outcomes rest in divine hands may prevent the helper from developing the toxic hyper-responsibility that drives cognitive collapse and severe compassion fatigue. A shared belief in a higher divine order (*Qadar*) might assist humanitarian workers in cognitively processing, organizing, and accepting highly traumatic events. This might be true for members from different religions. These beliefs are reinforced by community-based rituals, communal prayers, or gatherings that may serve as socially validated decompression in a stressful environment.

#### 5.2.4 Dance (Govend) and music (Lawj)

Secondary trauma alters the nervous system, trapping the helper in a state of somatic hyperarousal (anxiety, tension, sleeplessness). Traditional community practices such as *Govend* (synchronized circle dancing) and *Lawj* may function as somatic grounding exercises [see also (41)]. The rhythmic coordination, collective physical contact, and musical immersion might release somatic trauma retention, discharging accumulated stress from the helper's body far more effectively than isolated Western intellectual reflection. They might act as deeply embedded, culturally syntonic somatic valves and may contribute to the regulation of the nervous system helping reduce the physical and psychological tension accumulated during humanitarian crises. The students at IPP very often share their lunch breaks, sing together or have traditional picnics (S*eyran*).

#### 5.2.5 Newroz and collective celebrations as restart

On a broader societal level, collective celebrations and cultural symbolism provide essential avenues for psychological renewal. The celebration of *Newroz* (the Kurdish New Year on March 21st) symbolically represents the triumph of light over darkness, freedom, and the rebirth of nature. This event functions as an annual, collective moment of emotional catharsis that restores hope and psychological energy across the workforce. It represents a restart, where old things can be left behind.

#### 5.2.6 Community duties (Mevan)

Finally, the cultural mandate of radical hospitality and community duty (*Mevan*) fosters an intrinsic sense of purpose. Governed by traditional adages such as “*Mêvan mêvanê Xwedê ye*” (The guest is God's guest), providing aid to those in distress is elevated to the highest tier of moral honor and communal duty. This represents a deep cultural validation of the helper's identity and thereby generates immense intrinsic reward, serving as a potential powerful protective barrier against occupational devaluation and exhaustion.

## 6 Proposed solutions

### 6.1 What is needed for a healthy working environment?

This article discussed general and culturally specific protective factors against secondary traumatization. The empirical and practical foundation of these observations and proposed solutions relies partly on established literature but is mainly derived from the clinical training environment of the IPP in the KRI. These insights stem from regular case supervisions, student reports, discussions within lectures, and reflection meetings held with local staff and students within the last years.

#### 6.1.1 Personal cognitive and psychological factors

First of all, establishing clear emotional boundaries in relation to this challenging work is crucial for any psychotherapist. The psychological capacity to practice empathetic connection while maintaining a strict cognitive line between the client's trauma history and the helper's personal reality is essential. Professional realism and a realistic understanding of the limits of helping protect individuals from the toxic hyper-responsibility or “savior complexes” that rapidly drive secondary trauma. Active cognitive reframing by intentionally reappraising traumatic situations to focus on client resilience, survival, and small operational victories, rather than focusing solely on despair, helps prevent psychotherapists from becoming cynical. Utilizing specific, structured transitional rituals at the end of a shift (e.g., changing out of uniform, silent commuting, breathing exercises) can help to leave difficult stories of suffering and trauma at the workplace and thereby serve as a somatic and behavioral decompression strategy. Engaging in vigorous exercise, meditation, or other rhythmic activities helps to physically release the fight-or-flight energy and tension stored in the body from hearing traumatic stories. Cultivating a strong, non-work social identity and engaging in hobbies completely unrelated to social, medical, or humanitarian fields to restore cognitive balance is essential for maintaining long-term psychological resilience. In the region, many IPP students and therapists play soccer, go hiking in nature, go to the sauna, etc. or simply meet outside to walk through the bazaar.

#### 6.1.2 Healthy organizational and structural factors

As many young therapists in western therapy schools can start with rather “easy” to moderate case complexity, this is often not possible for inexperienced therapists in the KRI because of the high demand and the comparatively still existing low number of operating, highly skilled mental health professionals in the region. Still, as far as possible, a well dosed load diversification is crucial, i.e., maintaining a balanced mix of clients prevents helpers from being exposed exclusively to high-severity, graphic trauma narratives throughout the workday. Again, clinical supervision is essential. Regularly scheduled, reflective supervision sessions with an external expert provide a safe space to process countertransference and emotional residue before it solidifies into trauma. It creates a work environment that normalizes emotional vulnerability and allows helpers to report secondary distress early, without fear of being labeled incompetent or weak. Such a professional “safe haven” would also create a culture of psychological support and safety. It would counteract the burden of professional unclarity and therefore indirectly help protect and prevent mental health workers from additional psychological stress and mental disorders themselves.

For a long time, the IPP has prepared locally trained psychotherapists to step into this leading and supervising role. This requires that the local staff receive official recognition of having not only the duty but also legal authorization to do so. Currently, some local psychiatrist seem to have rather a strong interest in protecting their role, hindering the creation and establishment of a psychotherapy association. Such an association is crucial, as it will be able to fight for future rights of psychotherapists in the region. Establishing a psychotherapist law will not only make sure to have clarity of duties and responsibilities for psychotherapists, but it would also validate their important work. Table 4 depicts possible counter measures against culturally specific risk factors for secondary trauma for helpers in KRI.

**Table 4:** Culturally specific risk factors vs. risk alleviating factors in the Kurdistan Region of Iraq (KRI).

Culturally specific risk factor	Risk alleviating factors
*Over-identification & shared trauma* (*Hevpişkinî*) Shared history of regional trauma (e.g., Anfal, ISIS) triggers the helper's own unhealed wounds and blurs professional boundaries.	*Cognitive reframing & supervision* Using adaptive coping mechanisms and cognitive reappraisal; close supervision to process personal emotional triggers.
*Family collectivism (Xêzan)* Cultural obligation to absorb the emotional and financial distress of extended family; dense socialization deprives helpers of private spaces.	*Community networks & peer exchange* Building professional support networks; sharing the emotional burden with peers to normalize distressing reactions.
*Infinite sacrifice (Fidakarî)* Intense moral pressure to act with absolute self-sacrifice; setting professional boundaries is culturally viewed as a lack of compassion.	*Organizational support & boundary frameworks* Institutional rules that limit caseloads, regulate after-hours calls, and formally mandate practitioner self-care.
*Societal stigma & spiritual shame (Serm)* Suppressing secondary trauma symptoms to avoid being labeled “weak” or lacking faith (*Tawakul*), which accelerates severe PTSD.	*Structured intervision & safe spaces* Hierarchical-pressure-free case discussions among equals; cultivating a workplace culture of constructive error tolerance.
*Inter-tribal or inter-religious Countertransference* Deep internal conflict or historical guilt when treating individuals from specific clashing or victimized subgroups.	*Specialized competence & team debriefing* Specialized training in local sociopolitical realities; utilizing peer assistance to navigate moral injury and historical guilt.
*Facing moral dilemmas (e.g., treating ISIS terrorists)* Severe emotional rejection of perpetrators vs. the professional ethical belief that everyone deserves psychological treatment.	*Voluntary case takeover & close supervision* Ensuring all high-conflict cases are strictly optional; providing a safe space to share and guide intense emotional experiences.
*Project-specific hurdles in the KRI* High case severity early in careers, institutional resistance to a psychotherapy association, and lack of legal recognition.	*Structured mentoring & visible leadership* Providing intensive supervision for student therapists; organizational resource pooling and building of an association for psychotherapist; strong management commitment and lobbying for psychotherapists.

Observations are only partly based on existing literature and mainly derived from insights gained within the context of the specific clinical training of the IPP in the KRI.

### 6.2 Key challenges and proposed solutions within the framework of a culturally sensitive approach

Because some (former) students and at the IPP have personally experienced war and displacement, they are already at high risk of secondary traumatization, making self-care and supervision essential.

Besides, project specific risk factors arise because of the following reasons:

(a) High case severity: Student therapists at the IPP manage a relatively high volume of severe cases (under supervision) at the beginning of their careers.

(b) Institutional resistance: Local psychiatrists and political figures currently seem to hinder the creation of a psychotherapy association.

(c) Legal & bureaucratic hurdles: Psychotherapy is not yet officially recognized as a legal profession in the KRI, leading to a lack of national guidelines and unclear working conditions. This leads on the one hand to uncertainty concerning their professional and financial future. This unclear structure undermines the integrity of their helpers' professionalism by indirectly questioning their skills. On the other hand, it leads to a profound lack of self-efficacy amongst the helpers. As a consequence, if therapists don't experience a professional safe space, they ultimately struggle to provide the safe space for their clients, leading to worse therapy outcomes.

Some key aspects to alleviate the situation for the practicing therapists and lecturers include:

Cultural sensitivity: Including and fostering culturally specific protective factors against secondary traumatization.

Respecting cultural variety and winning over local authorities and religious leaders by including them in the process.

Empowering young therapists and lecturers via actively promoting young local staff to positions of authority explicitly demonstrating trust in their competence to counter traditional age-based hierarchies.

Providing supervision: Creating an “error-tolerant” environment that moves away from harsh, deficit-oriented exam styles toward emotional support and validation.

Providing professional and legal safety to Kurdish therapists with a psychotherapy association and official law for psychotherapy with professional nation-wide guidelines.

## 7 Summary and Conclusion

Secondary traumatization occurs when professional helpers absorb the traumatic imagery and narratives of clients, leading to PTSD symptoms like intrusive thoughts or hyperarousal. Interviewers, whether journalists, police officers, attorneys, or psychosocial workers, are themselves at risk of secondary traumatization when repeatedly exposed to the accounts of severely traumatized survivors (42). Decades of international research have identified robust, universal protective factors that insulate the helper's mind from this indirect distress (29). In the Kurdistan Region of Iraq (KRI), psychosocial work and mental health self-care are profoundly shaped by specific sociocultural, historical, and religious factors. Local aid workers are often members of the affected communities themselves.

Because mental health workers in post-war regions face a high risk of secondary traumatization, deliberate measures must be implemented to ensure that helpers are protected from becoming secondarily traumatized during their work. Within this context, the primary goal of the Institute for Psychotherapy and Psychotraumatology (IPP) is not only to provide direct psychological care but also to establish sustainable structures, recognizing that the psychological consequences of war often become fully visible only when considering their effects on indirectly affected communities and future generations. Besides, general risk alleviating factors against secondary traumatization, such as profound and error-tolerant supervision, peer support and self-care, cultural mechanisms serve as resilience factors for local psychotherapists, including cultural traditions and customs. This article highlighted such culturally specific protective factors with the example of Kurdish Culture, including among others community resistance, the unity of family, traditional customs, and the belief in destiny.

### 7.1 Limitations

The presented community case study presents a focused, practice-based account from a single institution, prioritizing qualitative depth over broad generalization. An inherent characteristic of this work is its reliance on localized, culture-bound clinical observations and unstructured accounts from the IPP, which naturally focuses its scope and limits its generalizability. Rather than seeking to establish statistical prevalence across the Kurdistan Region of Iraq (KRI), this close-range approach delivers a rich, phenomenological case study. This methodology captures the highly nuanced cultural dynamics and individual clinical experiences that are frequently obscured by macro-level data. Consequently, this specialized, ground-level perspective offers a valuable baseline and serves as a set of foundational hypotheses. Future research can build upon these insights by operationalizing the concepts through standardized, large-scale empirical testing and cross-cultural replication.

### 7.2 Implications for research, practice and political decision making

Further studies could investigate the extent to which culture-specific protective factors can be translated into other cultures and contexts, thereby serving as protective factors across diverse settings. Transcultural psychotherapy should not only adapt interventions to specific groups, but also holistically reflect the cultural context in which it takes place, incorporating local customs and traditions as health-promoting assets. This approach could facilitate the effective tailoring of intercultural psychotherapy programs and help overcome “west-is-best” attitudes. The long-term sustainability of the IPP requires full integration into the local health and education systems and the establishment of reliable local government funding mechanisms to ensure continuity beyond the period of international financial support. Only if local politicians and psychiatrists also support the idea and collaborate on equal footing, which includes the establishment of a psychotherapy association and official laws governing psychotherapy, will local psychotherapists encounter healthy working conditions within which they can reach the full range of their therapeutic potential. The study highlights that far-sighted policymaking - such as offering supportive conditions for healthcare providers - impacts the wellbeing of both patients and providers. Only when provider health is taken into account can long-term sustainability be achieved, paving the way for positive change over time. Aldamman et al. (20) argue that humanitarian organizations must prioritize preventing and mitigating psychological harm among staff and volunteers rather than just treating it. The same principle applies to psychotherapy training programs. Integrating these preventive frameworks into education ensures more robust, long-term societal protection that extends far beyond immediate crisis response.

## Data Availability

The original contributions presented in the study are included in the article/supplementary material, further inquiries can be directed to the corresponding author.

## References

[1] Dalenberg CJ Straus E Carlson EB. Defining trauma. In:Gold SN, editor. APA Handbook of Trauma Psychology: Foundations in Knowledge. Washington, DC: American Psychological Association (2017). p.15–33.

[2] Dashorst P Mooren TM Kleber RJ de Jong PJ Huntjens RJC. Intergenerational consequences of the Holocaust on offspring mental health: a systematic review of associated factors and mechanisms. Euro J Psychotraumatol. (2019) 10:1654065. doi: 10.1080/20008198.2019.1654065 31497262 PMC6720013

[3] Starrs CJ Békés V. Historical intergenerational trauma transmission model: a comprehensive framework of family and offspring processes of transgenerational trauma. Traumatology. (2025) 31:243. doi: 10.1037/trm0000505

[4] Yehuda R Lehrner A. Intergenerational transmission of trauma effects: putative role of epigenetic mechanisms. World Psychiatry. (2018) 17:243–57. doi: 10.1002/wps.20568 30192087 PMC6127768

[5] Acquadro Maran D Dolce V Colombo L. Editorial: secondary traumatic stress: risk factors, consequences, and coping strategies. Front Psychol. (2023) 14:1148186. doi: 10.3389/fpsyg.2023.1148186 36925604 PMC10011627

[6] El-Khalil C Tudor DC Nedelcea C. Impact of intergenerational trauma on second-generation descendants: a systematic review. BMC Psychol. (2025) 13:668. doi: 10.1186/s40359-025-03012-4 40597246 PMC12220155

[7] Ogińska-Bulik N Michalska P. Indirect trauma exposure and secondary traumatic stress among professionals: mediating role of empathy and cognitive trauma processing. Psychiatr Pol. (2024) 58:39–50. doi: 10.12740/PP/162930 38852183

[8] Sprang G Ford J Kerig P Bride B. Defining secondary traumatic stress and developing targeted assessments and interventions: lessons learned from research and leading experts. Traumatology. (2018) 24:95–100. doi: 10.1037/trm0000180

[9] Hama-Amin DS Mustafa AA Aziz EH. Mental health in the Kurdistan Region of Iraq. BJPsych Int. (2026) 23:1–3. doi: 10.1192/bji.2026.10100

[10] Mohammed I Ahmed H Hasin B Kizilhan J Nariman H Ahmed S . A study on long-term trauma-related mental health outcomes among Kurdish survivors of chemical attacks. Front Psychiatry. (2025) 16:1693072. doi: 10.3389/fpsyt.2025.1693072 41635518 PMC12862082

[11] Kizilhan J Ag Z. Collective and transgenerational trauma in psychotherapeutic treatment: implications for refugee and post-conflict contexts. Med Res Arch. (2026) 14:5. doi: 10.18103/mra.v14i5.7433

[12] Inter-Agency Standing Committee (IASC). IASC Guidelines on Mental Health and Psychosocial Support In Emergency Settings (2007). Available online at: https://interagencystandingcommittee.org (Accessed September 12, 2026).

[13] Skrodzka M Toruńczyk-Ruiz S Wnuk A. Gained social identification and sense of shared experience after flood exposure: psychosocial pathways to post-traumatic symptoms and growth. Appl Psychol Health WellBeing. (2026) 18:e70193. doi: 10.1111/aphw.70193 PMC13379722 42470196

[14] Kornwachs G Hautzinger M Kizilhan J. The importance of cultural understanding and practical solutions during the handover of a psychotherapy and psychotraumatology program in Northern Iraq into local hands. Front Psychiatry. (2024) 15:1434670. doi: 10.3389/fpsyt.2024.1434670 39119076 PMC11306170

[15] Bock C Heitland I Zimmermann T Winter L Kahl KG. Secondary traumatic stress, mental state, and work ability in nurses-results of a psychological risk assessment at a university hospital. Front Psychol. (2020) 11:298. doi: 10.3389/fpsyt.2020.00298 32395109 PMC7197484

[16] Rizkalla N Segal SP. Trauma during humanitarian work: the effects on intimacy, wellbeing and PTSD-symptoms. Euro J Psychotraumatol. (2019) 10:1679065. doi: 10.1080/20008198.2019.1679065 PMC6818129 31692832

[17] Cameron L McCauley M van den Broek N McCauley H. The occurrence of and factors associated with mental ill-health amongst humanitarian aid workers: a systematic review and meta-analysis. PLoS ONE. (2024) 19:e0292107. doi: 10.1371/journal.pone.0292107 PMC11095667 38748709

[18] Arpacioglu S Gurler M Cakiroglu S. Secondary traumatization outcomes and associated factors among the health care workers exposed to the COVID-19. Int J Soc Psychiatry. (2020) 67:84–9. doi: 10.1177/0020764020940742 32635786 PMC7348255

[19] Lemieux-Cumberlege AH Griffiths H Pathe E Burley A. Posttraumatic stress disorder, secondary traumatic stress, and burnout in frontline workers in homelessness services: risk and protective factors. J Soc Distress Homelessness. (2023) 33:329–40. doi: 10.1080/10530789.2023.2191405

[20] Aldamman K Bitar C Brichetto M Correnti M Jacome MC Chavarri CU. Beyond psychological support: an integrated approach to protecting the mental health of aid workers operating in armed conflict settings. Int Rev Red Cross. (2026) 108:1–19. doi: 10.1017/S1816383126101209

[21] Strohmeier H Scholte WF. Trauma-related mental health problems among national humanitarian staff: a systematic review of the literature. Euro J Psychotraumatol. (2015) 6:28541. doi: 10.3402/ejpt.v6.28541 26589256 PMC4654769

[22] Ballard E. Prevalence of Secondary Traumatic Stress Among Disaster Relief Volunteers Master's Thesis. Glassboro, NJ: Rowan University (2014).

[23] Shah SA Garland E Katz C. Secondary traumatic stress: prevalence in humanitarian aid workers in India. Traumatology. (2007) 13:59–70. doi: 10.1177/1534765607299910

[24] Community Peacemaker Teams. Three Months of Iran-Linked Attacks on the Kurdistan Region of Iraq (2026). Available online at: https://cpt.org/2026/06/04/three-months-of-iran-linked-attacks-on-the-kurdistan-region-of-iraq (Accessed August 29, 2026).

[25] Kizilhan JI. Transcultural Psychiatry: A Practice Manual for Psychotherapists and Traumatologists. Bonn: VWB Verlag für Wissenschaft und Bildung (2020).

[26] Kizilhan JI Friedl N Neumann J Traub L. Potential trauma events and the psychological consequences for Yazidi women after ISIS captivity. BMC Psychiatry. (2020) 20:256. doi: 10.1186/s12888-020-02671-4 PMC7247249 32448135

[27] Mustafa SJ. Social-psychological consequences of post traumatic stress disorder. Koya Univ J Hum Soc Sci. (2025) 29:88–101. doi: 10.14500/kujhss.v8n1y2025.pp82-92

[28] Kizilhan JI Weigelt A. Healing after genocide: a cross-sectional study on emotion-focused coping and psychosocial recovery among Yazidi survivors. Med Res Arch. (2025) 13:7. doi: 10.18103/mra.v13i7.6772

[29] Whittenbury K Clark SL Brooks M Murphy T Turner MJ Fawcett H. Strengths for helping professionals exposed to secondary trauma: a scoping review. Trauma Violence Abuse. (2025) 26:251–64. doi: 10.1177/15248380241309371 40022616 PMC11872055

[30] Collett G Emad A Gupta AK. The impact of workplace support components on the mental health and burnout of UK-based healthcare professionals: insights from the CoPE-HCP cohort study. Clin Med. (2025) 25:100324. doi: 10.1016/j.clinme.2025.100324 PMC12153366 40334943

[31] Padmanabhanunni A Pretorius TB. Cognitive adaptation to stress and trauma: the role of self-appraised problem-solving in posttraumatic stress disorder. Chronic Stress. (2023) 7:24705470231189980. doi: 10.1177/24705470231189980 37520891 PMC10375036

[32] Karina TA Pratitis NT. Relationship between work-life balance, role ambiguity, and burnout in Generation Z. J Sci Res Educ Technol. (2025) 4:647–59. doi: 10.58526/jsret.v4i1.726

[33] Soltania I Hajatpourb S Khorramb J Nejatic MH. Investigating the effect of role conflict and role ambiguity on employees' job stress: articulating the role of work-family conflict. Manag Sci Lett. (2013) 3:1927–36. doi: 10.5267/j.msl.2013.06.036

[34] Shanafelt TD Gorringe G Menaker R Storz KA Novotny D Buskirk SJ . Impact of organizational leadership on physician burnout and satisfaction. Mayo Clinic Proc. (2015) 90:432–40. doi: 10.1016/j.mayocp.2015.01.012 25796117

[35] West CP Dyrbye LN Erwin PJ Shanafelt TD. Interventions to prevent and reduce physician burnout: a systematic review and meta-analysis. Lancet. (2016) 388:2272–81. doi: 10.1016/S0140-6736(16)31279-X 27692469

[36] Sutton L Rowe S Hammerton G Billings J. The contribution of organisational factors to vicarious trauma in mental health professionals: a systematic review and narrative synthesis. Euro J Psychotraumatol. (2022) 13:2022278. doi: 10.1080/20008198.2021.2022278 35140879 PMC8820814

[37] National Child Traumatic Stress Network. NCTSN Trauma-Informed Organizational Assessment (TIOA). UCLA-Duke University National Center for Child Traumatic Stress (2020). Available online at: https://www.regionalcacs.org/wp-content/uploads/2022/12/NCTSN_secondary-traumatic-stress-core-competencies-for-trauma-informed-support-and-supervision-cross-disciplinary-version-companio.pdf (Accessed September 12, 2026).

[38] Butt MK Eimontas J Grigutyte N Amin R. Interventions for secondary traumatic stress among mental health professionals serving trauma-affected populations: a systematic review. BMC Psychol. (2026) 14:112–28. doi: 10.1186/s40359-026-04753-6 42174636 PMC13374317

[39] Butt MK Grigutyte N Eimontas J. Protective and risk factors for STS among mental health professionals serving war refugees in Lithuania and Pakistan. Euro J Psychotraumatol. (2025) 16:2543205. doi: 10.1080/20008066.2025.2543205 40833378 PMC12372480

[40] Yaqeen Institute for Islamic Research. Predestination vs. Free Will in Islam: Understanding Allah's Qadr (2017). Available online at: https://yaqeeninstitute.org/read/paper/predestination-vs-free-will-in-islam-understanding-allahs-qadr (Accessed September 12, 2026).

[41] Silva F Dokter D Samaritter R. Grounding in dance movement therapy for people with persistent physical complaints. Body Movement Dance Psychotherapy. (2026) 21:184–99. doi: 10.1080/17432979.2025.2555226

[42] Kizilhan J. Interviews with survivors of catastrophic events: possibilities, risks, and culturally sensitive perspectives. MEDIRES. (2025) 4:1–9. doi: 10.58489/2836-3558/037

